# Psychometric evaluation of the postpartum specific anxiety scale – research short-form among Iranian women (PSAS-IR-RSF)

**DOI:** 10.1186/s12884-023-05855-4

**Published:** 2023-07-21

**Authors:** Sepideh Mashayekh-Amiri, Mohammad Asghari Jafarabadi, Siân M Davies, Sergio A. Silverio, Victoria Fallon, Maryam Montazeri, Mojgan Mirghafourvand

**Affiliations:** 1 Students Research Committee, Midwifery Department, Faculty of Nursing and Midwifery, Tabriz University of Medical sciences, Tabriz, Iran; 2 Cabrini Research, Cabrini Health, Malvern, VIC 3144 Australia; 3 School of Public Health and Preventative Medicine, Faculty of Medicine, Nursing and Health Sciences, Monash University, Melbourne, VIC 3800 Australia; 4 Road Traffic Injury Research Center, Tabriz University of Medical Sciences, Tabriz, Iran; 5 School of Psychology, Liverpool John Moores University, Byrom Street, Liverpool, Merseyside, L3 3AF UK; 6 Department of Women & Children’s Health, School of Life Course & Population Sciences, King’s College London, London, SE1 7EH UK; 7 Department of Psychology, Institute of Population Health, University of Liverpool, Liverpool, L69 7ZA UK; 8 Midwifery Department, Faculty of Nursing and Midwifery, Tabriz University of Medical sciences, Tabriz, Iran; 9 Social Determinants of Health Research Center, Department of Midwifery, Faculty of Nursing and Midwifery, Tabriz University of Medical Sciences, Tabriz, Iran; 10 Menopause Andropause Research Center, Ahvaz Jundishapur University of Medical Sciences, Ahvaz, Iran

**Keywords:** Postpartum anxiety, Short-form, PSAS-IR-RSF, Mental health, Iran, Psychometrics

## Abstract

**Background:**

The increasing prevalence of postpartum anxiety as a common psychological problem affects a large part of women’s lives. Despite the existence of tools in this field, but due to the lack of specificity in reflecting postpartum anxiety, it is necessary to have a specific tool to screen it. Since the psychometric evaluation of the Postpartum Specific Anxiety Scale-Research Short-Form (PSAS-RSF) among Iranian women has not been assessed in Iran until now, so we decided to conduct this study with the aim of psychometric evaluation of the PSAS-IR-RSF.

**Methods:**

We included 180 women (six weeks to six months postpartum) in the study by random sampling during the period from December 2021 to June 2022. We examined the validity of the PSAS-IR-RSF tool in terms of face, content and construct (through exploratory and confirmatory factor analyses). We used internal consistency and test-retest reliability to determine the reliability of the scale.

**Results:**

In the present study, content validity index (CVI) and content validity ratio (CVR) of the PSAS-IR-RSF tool were equal to 0.91 and 0.97, respectively. We extracted a four-factor structure through the process of exploratory factor analysis. The values of fitting indices confirmed the validity of the model. Cronbach’s alpha coefficient was equal to 0.72 and intra-class correlation coefficient (with 95% confidence interval) was 0.97 (0.98 to 0.93).

**Conclusions:**

The Persian version of the PSAS-IR-RSF is a valid and reliable tool for the specific evaluation of postpartum anxiety among Iranian women.

## Background

Although pregnancy and childbirth periods are often considered to be a unique experience in the life of most women, it can become a stressful period due to the occurrence of physiological and psychological changes. Consequently, women will face many problems due to the lack of adaptation to these changes during pregnancy and childbirth and the change in their role in the family and society [1].

One of the common psychological problems that occur in the postpartum period is anxiety. Anxiety is an unreasoned and unjustified fear, unreasonable tension or worry with common signs of doubt, uncertainty, helplessness, and physiological emotions that may cause various psychological, cognitive, emotional, and physical difficulties [2]. The result of a systematic review and meta-analysis in 2022, reported the prevalence of self-reported anxiety symptoms in antenatal and postnatal period as 29.2% and 24.4%, respectively. Additionally, the prevalence of clinically-diagnosed anxiety disorder in antenatal and postnatal period was reported as 8.1% and 16.0%, respectively [3].

The occurrence of postpartum anxiety is more common than postpartum depression and may be co-morbid with symptoms of postpartum depression [4]. Postpartum anxiety disorder is clinically a disproportionate and overwhelming worry leading to functional impairment. Physical anxiety symptoms include fatigue, irritability, difficulty concentrating, and sleep disorders [5]. Although these symptoms are easily recognizable, these are sometimes ignored on go unrecognized due to the challenge of differentiating between what is normal; and adaptive anxiety as compared to those which are maladaptive symptoms in the postpartum period, leading to a delay in diagnosis or complete misdiagnosis [6].

Risk factors for postpartum anxiety are demographic characteristics, income level, lack of pain control during labor [7], breastfeeding difficulties [8], poor physical health [9, 10], hormonal changes, previous infertility [8], insufficient social skills [7], low maternal self-efficacy [7, 10], lack of social support and low partner support [7, 9, 10], history of psychological difficulties, negatively experienced perception of childbirth [10], unwanted pregnancy [8], and the level of mother’s education [7, 8, 10].

The event of postpartum anxiety provides the ground for the occurrence of problems for the mother, newborn, and other family members [11]. Such a situation may negatively impact the level of attachment between the mother and the newborn and other family relationships and even threaten the security and health of the mother, newborn, and other children [12].

Postpartum anxiety is associated with wide consequences for the mother and thenewborn. Decreased quality of life, increased risk of chronic diseases [7], infant temperament and behavioral problems [13], adverse outcomes of infant feeding [7, 14], impaired interactions with spouse and child [15], decreased self-efficacy of mothers [16], poor mental and cognitive development of infant [7, 16], and economic burden on the health system are some examples of consequences [17].

To deal with these consequences, we must prioritise the importance of correct and timely screening and identification of anxieties during the postpartum period [18]. Previously, researchers used scales such as the State-Trait Anxiety Inventory (STAI) and General Anxiety Disorder-7 Questionnaire (GAD-7) to measure postpartum anxiety [19, 20]. However, since these tools are designed for the general adult population, it is not easy to use them in postpartum period, and on the other hand, these general scales cannot specifically reflect mothers’ anxiety [21, 22]. Consequently, low scores on these tools do not indicate the absence of problems or symptoms [23].

Fallon et al. designed a Postpartum Specific Anxiety Scale to overcome the problems of general tools for measuring postpartum anxiety; it has 51 questions in the form of a four-option Likert scale, which includes four domains. Its components include anxieties about psychosocial adjustment to motherhood, practical infant care, maternal competence and attachment, and infant safety and welfare anxieties [24].

The PSAS has been translated and validated globally [25] including a version of the long form into Persian [26]. Although this tool has an acceptable capacity to measure postpartum anxiety; following repeated requests for a short form, Davies et al. designed the short form PSAS-IR-RSF in 2021 with 16 questions in the form of a four-point Likert scale [27]. This tool has been acceptable and widespread; the relevant team in Iran has validated the long form of this scale [26]. Since the long form has evidence for validity and reliability which is also becoming apparent for the research short form we decided to conduct this study with the aim of psychometric evaluation of the Postpartum Specific Anxiety Scale-Research Short-Form among Iranian women (PSAS-IR-RSF).

## Study aim and design

This cross-sectional study was carried out to test the psychometric properties of the Postpartum Specific Anxiety Scale-Research Short-Form (PSAS-IR-RSF) among Iranian women.

### Study participants and sampling

This was a study with 180 women in the postpartum period between six weeks and six months postpartum, with health records in the health centers of Tabriz-Iran.

The inclusion criteria for the study included women who gave birth with a full-term newborn within six weeks to six months after a vaginal or caesarean delivery. Women were excluded from the study if they did not complete more than 20% of the questions in the questionnaire; and did not have a history of a traumatic event in the past six months, including the death of a relative.

The sample size required to perform factor analysis is 5 to 10 participants per question [28], which considering 16 items and five people per item, it is 80 participants. However, according to the cluster sampling method and by applying the design effect equal to two, the sample size increased to 160 participants, and taking into account about 10% possible attrition, 180 participants were examinable.

### Recruitment

The sampling method was random cluster. Thus, in the first stage, we used the website www.random.org and selected a quarter of centers from among 82 health centers in Tabriz. Then we listed the mothers who spent six weeks to six months postpartum, based on the integrated health system (SIB system in Farsi), determined the number of selected women from each center in proportion to the sample size and selected women from the center randomly, again using the website www.random.org.

The researcher contacted individuals using their phone numbers and gave them brief explanations about the reasons, quality, and process of the research. If desired, we requested the mother to visit the covered health center on a certain date and time for further explanations and to complete the questionnaires. After the referral, the researcher studied the participant first in terms of basic information and inclusion and exclusion criteria. If they had the eligibility criteria of the research, she provided them with comprehensive information about the reasons for conducting the research, the benefits, the results, and the confidentiality of the information and the research process. If they agreed to participate in the research, they completed the informed consent form of participation, and the researcher provided them with self-administered questionnaires. The questionnaires provided to the mothers included those of recording socio-demographic and obstetric information, and the Postpartum Specific Anxiety Scale – Research Short-Form (in Persian).

### Instruments

#### Socio-demographic and obstetrics checklist

This checklist includes questions about age, gestational age at delivery, birth weight of the newborn, gender of the newborn, level of education, occupation, income status, desired or unwanted pregnancy, and mode of birth.

#### Postpartum specific anxiety scale-research short-form questionnaire [PSAS-RSF

Davies et al. [27] developed this tool in 2021 in the United Kingdom. It contains 16 items and has four dimensions: anxiety about psychosocial adjustment to motherhood, anxiety of practical infant care, anxiety of maternal competence and attachment, infant safety and welfare anxieties (each dimension has 4 questions). It is a 4-point Likert scale (from Not at all = 1 to almost always = 4). It is a shortened form of the measurement tool of the Postpartum Specific Anxiety Scale in 2016 designed by Fallon et al. It includes 51 questions as a 4-point Likert, and its four dimensions have 15, 11, 7, and 18 items, respectively; which have been previously psychometrically evaluated by the research team in Iran in 2021 [26]. The minimum score on the Research Short Form (PSAS-IR-RSF) is 16, and the maximum score is 64 [27].

#### Precedure

We prepared the Persian version of the tool (PSAS-IR-RSF) through several steps, including translation of the tool, face validity, content validity, evaluation of construct validity and its reliability.

#### Translation process

As for translation, at first, after obtaining permission to translate the present questionnaire from its designers [27], the translation of the original version was done by the forward-backward method. Two translators fluent in English: (1) whose mother tongue was Persian; and (2) were familiar with the concept of postpartum anxiety, translated its English form into Persian independently. Then the two translators discussed the contradictions in their translation and prepared the Persian version after correcting the contradictions and combining the two translations. Then, this version was given to an English-speaking translator who is fluent in the Persian language to translate this Persian version into English, and finally, the two versions forward-backward and the original version were compared; in case of inconsistency between these two versions, the necessary corrections were made through referring to the Persian version [29].

#### Face validity

Both qualitative and quantitative methods were appropriate for face validity. In the qualitative method, the opinions of ten experts with knowledge and experience in postpartum anxiety and tool development commented on the level of simplicity, transparency, and relevance of the items. These cases were modifiable in terms of using appropriate, clear words, grammar, and the importance of cases in Iran based on their context. As for the quantitative method, 30 eligible women responded to the PSAS-IR-RSF and rated the level of importance of the items from 5 (extremely important) to 1 (not at all important). Then face validity was quantitatively measured through the item impact method based on women’s opinions [30]. The researcher calculated the impact score of each item according to the answers chosen by women, based on the following formula (Impact Score = Frequency (%) × Importance); (Frequency: percentage of 4 and 5 responses and Importance: average responses given to the item.) Impact Score is confirmable with a score above 1.5 [31].

### Content validity

The coefficient of the content validity ratio (CVR) and the content validity index (CVI) was obtainable for confirming the validity of the content based on the opinions of ten experts (in midwifery and reproductive health). The researcher designed a checklist with two sections for each expert. The first and second parts of the checklist were about calculating CVI and CVR, respectively. The first part of the checklist evaluated the clarity, simplicity, and relevance of the item based on a 4-point Likert scale. The second part evaluated the necessity of each item based on a 4-point Likert scale from not useful to necessary. CVR higher than 0.62 and CVI higher than 0.79 were supposedly valid [32].

### Construct validity

Construct validity refers to the consistency between measurement and theoretical concepts. In other words, construct validity evaluates the appropriateness of the scale to measure whether the scale items can support the theoretical and operational definitions of a concept. Construct validity is always about the question: *“Which construct does the scale measure?“* [33]. Exploratory factor analysis (EFA) and confirmatory factor analysis (CFA) methods were appropriate for determining construct validity.

#### Exploratory factor analysis

The factors for exploratory factor analysis were extracted after calculating the correlation matrix between the variables (through the principal axis factoring method, followed by direct oblimin (to examine the relationship between factors). Each factor was named based on the variables (questions), and the compatibility of these factors with the concept and dimensions of anxiety was examined. Kaiser-Meyer-Olkin (KMO) was appropriate for investigating the adequacy of the model, Bartlett’s test to check the sphericity and the variance index expressed by the factors and the total, the Eigenvalue method for determining the number of factors, and cut-off point 0.3 for assigning the factor parameters (correlation between the questions and the factors) [34].

#### Confirmatory factor analysis

Confirmatory factor analysis evaluated the structure of factors extracted from exploratory factor analysis. The fitting of indices evaluated the proportion of the exploratory model. Root Mean Square Approximation (RMSEA) less than 0.08, Approximation Square Mean Square Root Standardized (SRMSEA) < 0.08, Index Fit Index (CFI) ≥ 0.90, normed Chi2 < 5, Index Tucker-Lewis (TLI) ≥ 0.95 were supposed to confirm the model. The confirmatory factor analysis clarified the significance of the model coefficients test and the correlation test between the factors [34].

### Reliability

Test-retest reliability and internal consistency were applicable to determine the reliability of the questionnaire (The interval between two tests should be such that, on the one hand, the forgetting of the questionnaire questions does occur, and on the other hand, the change in the desired phenomenon does not occur; this interval is two weeks to one month). Thirty randomly selected mothers completed the questionnaire. The intra-class correlation coefficient (ICC) and its 95% confidence interval (95% CI) (obtained from answering the questionnaire twice) were calculated for the entire questionnaire. Cronbach’s alpha coefficient was used for the entire tool in order to determine internal consistency. Its purpose is to check the correlation between the variables that make up the desired structure or scale. The present study considered Cronbach’s alpha coefficient higher than 0.7 [35].

### Statistical analysis

All analyses were conducted using IBM SPSS Statistics 22 (IBM Corp, Armonk, NY, USA) and STATA 14 (Statcorp, college station, Texas, USA). Data were expressed using Mean (SD) for numeric variables and frequency (percent) for categorical variables.

### Ethical considerations

This study was approved by the Ethics Committee of the Tabriz University of Medical Sciences, Tabriz, Iran (Ethics code: IR.TBZMED.REC.1400.487). Before using the PSAS-IR-RSF, the required permission was obtained from the PSAS Working Group via email. During the study, written consent was obtained from all participants. Participants were assured their information and names would be kept confidential in the results reported. It was also be explained that they could withdraw from the study at any stage of the study.

## Results

### Participant characteristics

From the 82 health centers in Tabriz, we randomly included 180 mothers in the present study (December 2021 and June 2022). The mean age (SD) of the participants was 27.6 (5.8) years, and more than three-quarters of them (93.9%) were housewife (Table 1).

**Table 1:** Characteristics of the study participants (n = 180)

Characteristics	N (%)
**Age** (Year)^*^	27.6 (5.8)
**Gestational ag**e (Week)^*^	37.9 (2.0)
**Baby’s weight** (Gram)^*^	3187.9 (514.8)
**Baby’s gender**	
Male	96 (53.3)
Female	84 (46.7)
**Education**	
Intermediate or below	55 (30.5)
High school or diploma	125 (69.4)
**Job**	
Housewife	169 (93.9)
Employee	11 (6.1)
**Income**	
Not at all sufficient	35 (19.4)
Relatively sufficient	110 (58.2)
Completely sufficient	35 (19.4)
**Type of delivery**	
Normal vaginal delivery (NVD)	48 (26.7)
Caesarean section (C/S)	132 (73.3)
**Unwanted pregnancy**	
Yes	151 (83.9)
No	29 (16.1)

^*^_The numbers were reported as mean (standard deviation)_

### Face and content validity

As for face validity, all items were appropriate and without ambiguity or difficulty; receiving a minimum impact score of 1.5. As for the content validity, all items obtained the minimum acceptable value of CVI and CVR, which were 0.91 and 0.97, respectively (Table 2).

**Table 2:** The impact score, CVI, and CVR for questions (n = 10 Expert)

Items		Impact score	CVI	CVR
1		4.00	1.00	1.00
2		4.00	0.90	1.00
3		4.00	0.86	1.00
4		4.00	0.76	1.00
5		4.00	1.00	1.00
6		4.00	0.93	1.00
7		4.00	0.93	1.00
8		4.00	0.93	1.00
9		4.00	0.90	1.00
10		4.00	1.00	0.80
11		4.00	0.96	1.00
12		4.00	1.00	1.00
13		4.00	0.93	1.00
14		4.00	0.70	0.80
15		4.00	0.93	1.00
16		4.00	0.86	1.00

CVI: Content Validity Index, CVR: Content Validity Ratio

### Construct validity

As for the construct validity, the KMO value was 0.68 through exploratory factor analysis at a significant level of less than 0.001. The larger KMO was equal to 0.7, and the significant result of Bartlett’s test confirmed the adequacy of the model. Consequently, the study obtained a 4-factor structure with a total variance of about 30.3%. The first factor includes the anxieties of the psychosocial adjustment to motherhood with 4-items. The second factor includes the anxieties of the practical infant care with 4-items. The third factor contains the anxieties of maternal competence and attachment with 3-items. Finally, the fourth factor contains infant safety and welfare anxieties, with 4-items. Question 10 [I have had negative thoughts about communication with my child] was removed due to a factor loading of less than 0.3 (Table 3).

**Table 3:** Facture structure of the PSAS-RSF

Scale item	Factor 1	Factor 2	Factor 3	Factor 4
**Factor 1: Psychosocial adjustment to motherhood**				
1. I have felt that I have had less control over my day than before my baby was born	0.697			
2. I have felt unable to juggle motherhood with other responsibilities	0.490			
3. I have worried that I am not going to get enough sleep	0.618			
4. I have worried more about my finances than before my baby was born	0.323			
**Factor 2: practical infant care anxieties**				
5. I have worried about my baby’s milk intake		0.643		
6. I have worried about my baby’s weight		0.788		
7. I have worried about the length of time by baby sleeps		0.603		
8. I have worried about getting my baby into a routine		0.346		
**Factor 3: maternal competence and attachment anxieties**				
9. I have felt that my baby would be better cared for my someone else			0.311	
10. I have worried I will not know what to do when my baby cries			0.727	
11. I have worried that my baby is picking up on my anxieties			0.384	
**Factor 4: infant safety and welfare anxieties**				
12. I have repeatedly checked on my sleeping baby				0.579
13. I have worried that my baby will stop breathing while sleeping				0.536
14. I have felt frightened when my baby is not with me				0.551
15. I have worried about my baby being accidentally harmed by someone or something else				0.579
% Variance Explained	7.58	9.49	5.31	7.88
Total variance	30.25			
Cronbach’s alpha for total scale	0.72			
Intraclass Correlation Coefficient (95% CI)	0.93 (0.86 to 0.97)	0.97 (0.93 to 0.98)	0.91 (0.82 to 0.96)	0.95 (0.90 to 0.98)
Total scale	0.97 (0.93 to 0.98)			
Mean (SD)	3.5 (2.5)	3.4 (2.7)	1.6 (1.5)	5.5 (3.1)
Total score	14.3 (8.0)			

**Fig. 1:**
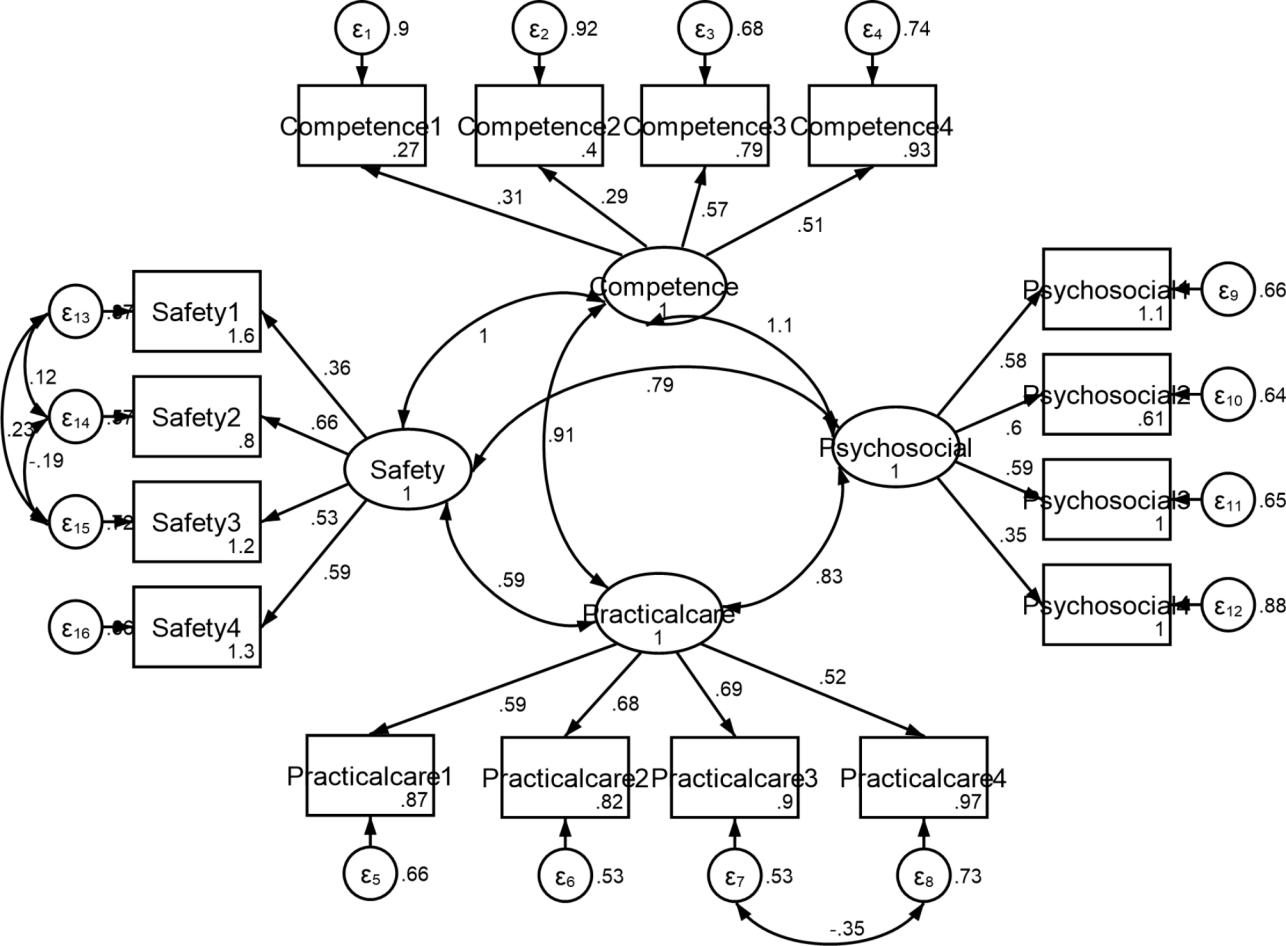
CFA factor loading of the PSAS-IR-RSF All factor-item relationships were significant (P < 0.05)

As for confirmatory factor analysis, x^2^/df was 1.521, and the RMSEA index value was 0.054, which confirmed the validity of the model. TLI and CFI fitting indices were equal to 0.9. Consequently, this model has achieved a favorable level of fitting, based on which we can confirm its factorial structure (Table 4). Figure 1 shows a path diagram with standard coefficients of confirmatory factor analysis for the considered conceptual model.

**Table 4:** Confirmatory factor analyses fit Index of the PSAS-RSF (n = 180)

Fit Indices	Value
χ2	143.019
P	˂0.001
$$\raisebox{1ex}{${x}^{2}$}\!\left/ \!\raisebox{-1ex}{$df$}\right.$$	1.521
CFI	0.920
SRMR	0.060
TLI	0.898
RMSEA [90% CI]	0.054 (0.035, 0.071

χ2: chi-square; df: degrees of freedom; χ2/df: normed chi-square; RMR: Root Mean R; RMSEA, root mean square error of approximation; CFI: Comparative Fit Index; SRMR: Standardized root mean squared residual; TLI: Tucker–Lewis inde

### Reliability

As for the reliability of the tool, Cronbach’s alpha coefficient calculated for the tool was equal to 0.72, which indicates the good internal consistency of the questionnaire. The ICC (95% CI) was 0.97 (0.98 to 0.93) for the test-retest reliability (Table 3).

## Discussion

Even though the birth of a newborn is an exciting moment for most mothers, the postpartum period can be associated with many challenges from the point of view of mental health. Although it is normal to experience mild degrees of anxiety in response to new motherhood and the birth of a newborn, the anxiety experienced by some mothers is excessive and debilitating, disrupting the natural process of their life and depriving them of the joy of this period [17].

Neglecting women during the postpartum period is associated with the increasing prevalence of postpartum anxiety and irreparable consequences for the mother and the newborn, which requires prevention through the identification of this disorder and its measurement with valid and reliable tools [36].

Unfortunately, the treatment rates of postpartum anxiety have been reportedly low, which indicates a failure to correctly identify this disorder and the lack of valid tools to measure postpartum anxiety [37]. Therefore, the present study aimed at psychometric evaluation of the Postpartum Specific Anxiety Scale-Research Short-Form among Iranian women (PSAS-IR-RSF).

The study results indicated that the Persian version of this scale is a valid and reliable tool for evaluating postpartum anxiety among Iranian women. This questionnaire is shortened version of the postpartum anxiety tool designed by Fallon et al., and validated psychometrically in Iran [26]. The Research Short Form was developed for greater ease, greater acceptability, and easier completion and access, and until today, it has been highly regarded by various countries, with several validation studies underway.

Content validity (quantitative and qualitative), face validity (qualitative and quantitative), and construct validity (exploratory and confirmatory factor analysis) determined the PSAS-IR-RSF’s validation in order to determine the psychometric properties of this tool. Internal consistency (Cronbach’s alpha coefficient) determined the reliability of the tool. Cronbach’s alpha coefficient obtained for the scale was equal to 0.72, which indicates its good internal consistency. In this regard, Davies et al. reported in a study that the reliability for the whole scale was 0.96 and for its four factors in the range to be 0.78–0.90 [27].

Factor analysis is an important statistical tool to confirm the validity of questionnaires. During the exploratory factor analysis, the researcher obtained a 4-factor structure corresponding to the factors of the original version for 15 questions of the questionnaire, and the explained variance of the factors for measuring the desired concept in the questionnaire was about 31% for the 4-factor structure, which was in the original questionnaire equal to 44% [27]. The value of KMO, the significance of Bartlett’s test, and the value of RMSEA (0.054) also confirmed the adequacy of the model.

The first factor obtained during the exploratory factor analysis is the anxieties of the psychosocial adjustment to motherhood, which includes 4 items; it concerns the mother’s adaptation after the birth of the newborn regarding the management of personal appearance, relationships and social support, work, mother’s finance, and sleep. The second factor is anxieties of practical infant care, including 4 items that deal with anxieties of newborn care such as feeding, sleep, and routine. The third factor concerns the anxieties of maternal competence and attachment with 3 items and deals with anxieties of mother’s self-efficacy, parental competence, and mother-newborn relationship. Finally, the fourth factor expresses the anxiety about the infant safety and welfare, which includes 4 items and is about fear of newborn diseases, accidents, and newborn death [27].

The factors extracted from the questionnaire align with the results of some studies conducted in this field. During interviews with mothers after childbirth, Brockington et al. reported anxiety as moderate in 43% and severe in 14%. The most common themes included fear of the death of the newborn (32%), fear of others’ criticism about failure to play properly the role of mother (19%) and fear of lack of support from the partner (16%) [38].

One of the main factors addressed in most studies is the issue of social support. As the study by Cena et al. showed in 2021, the risk of anxiety is significantly higher in mothers who had depression or anxiety during pregnancy and did not have enough psychological support from their spouses [39]. Likewise, the results of a study by van den Berg et al. in 2021 showed that factors associated with a higher risk of postpartum anxiety include higher education level, history of depression, premature birth, negative childbirth experience, excessive crying of the newborn, low self-efficacy of the mother, mother’s current weak health and low partner support [10]. Not receiving social support from the partner has a negative effect on women’s emotional state. Not paying attention to women’s needs and feelings reduces their self-confidence and increases by three times the risk of postpartum depression and anxiety in women without a history of postpartum depression [40].

Two other factors are the anxieties of the practical infant care and its safety and welfare. In this regard, the results of a review study carried out by Field in 2017 showed that the factors of postpartum anxiety could fall into four categories, including demographic factors, childbirth experiences, social support, and history of psychological problems. Demographic risk factors for postpartum anxiety include the mother’s youth, high education level and being employed, childbirth experiences including being a primiparous child, cesarean section, fear of birth and fear of death during childbirth, lack of control during childbirth, and low self-efficasy for childbirth and anxiety of taking care of the newborn. Social support problems include lack of family support, conflicts in a marital relationship with the spouse, social health issues, and psychiatric history problems, including depression and anxiety before pregnancy [7].

A study formed focus groups and conducted individual interviews with 105 women in rural Nepal through a grounded theory approach to conceptualize postpartum anxiety as ‘tension’. It showed that anxiety during this period focuses on factors like the safety and well-being of the newborn, caring for the newborn and the perceived inability to play the role of a perfect and competent mother from society’s perspective [41].

Another important factor in line with the present study, and other studies have addressed it, are the mother’s self-efficacy and sense of competence in playing the role of a mother. Many women in the postpartum period feel that they do not fulfill their role as mothers properly and are not confident about their abilities, leading to anxiety. As researchers have suggested, a significant discrepancy between women’s expectations and beliefs and their actual experiences of motherhood may cause feelings of anxiety in the postpartum period [42].

### Strengths and limitations

One of the strengths of this study is the random selection of participants from women who have given birth and the inclusion of women with a history of vaginal delivery and cesarean section. Conducting psychometrics of the PSAS-IR-RSF scale for the first time in Iran is another strength of our study. Performing CFA and EFA on a set of data can be mentioned as a limitation of the present study.

## Conclusion

The PSAS-IR-RSF scale is a valid and reliable tool for evaluating postpartum anxiety. This scale is recommendable due to its specificity for the postpartum period and its shortness, ease of completion, greater acceptability, and easier access during the postpartum period. Future studies with a larger sample size and conducting exploratory and confirmatory factor analysis on two separate samples, as well as psychometric evaluation of this scale in different contexts, will be very helpful.

## Data Availability

The datasets used and/or analyzed during the current study are available from the corresponding author upon reasonable request.

## Abbreviations

PPA: Postpartum anxiety
PSAS-IR-RSF: Postpartum Specific Anxiety Scale-Research Short-Form
EFA: Exploratory factor analysis
CFA: Confirmatory factor analysis
ICC: Intraclass Correlation Coefficient
SD: Standard deviation
CVI: Content validity index
CVR: Content validity ratio
KMO: Kaiser-Meyer-Olkin
RMSEA: Root mean squared error of approximation
CFI: Comparative fit index
TLI: Tucker–Lewis index
