# Correction: Psychometric evaluation of the postpartum specific anxiety scale – research short-form among Iranian women (PSAS-IR-RSF)

**DOI:** 10.1186/s12884-023-05883-0

**Published:** 2023-08-03

**Authors:** Sepideh Mashayekh-Amiri, Mohammad Asghari Jafarabadi, Siân M. Davies, Sergio A. Silverio, Victoria Fallon, Maryam Montazeri, Mojgan Mirghafourvand

**Affiliations:** 1 Students Research Committee, Midwifery Department, Faculty of Nursing and Midwifery, Tabriz University of Medical Sciences, Tabriz, Iran; 2 Cabrini Research, Cabrini Health, Malvern, VIC 3144 Australia; 3 School of Public Health and Preventative Medicine, Faculty of Medicine, Nursing and Health Sciences, Monash University, Melbourne, VIC 3800 Australia; 4 Road Traffic Injury Research Center, Tabriz University of Medical Sciences, Tabriz, Iran; 5 School of Psychology, Liverpool John Moores University, Byrom Street, Liverpool, L3 3AF Merseyside UK; 6 Department of Women & Children’s Health, School of Life Course & Population Sciences, King’s College London, London, SE1 7EH UK; 7 Department of Psychology, Institute of Population Health, University of Liverpool, Liverpool, L69 7ZA UK; 8 Midwifery Department, Faculty of Nursing and Midwifery, Tabriz University of Medical Sciences, Tabriz, Iran; 9 Social Determinants of Health Research Center, Department of Midwifery, Faculty of Nursing and Midwifery, Tabriz University of Medical Sciences, Tabriz, Iran; 10 Menopause Andropause Research Center, Ahvaz Jundishapur University of Medical Sciences, Ahvaz, Iran

**Correction: BMC Pregnancy Childbirth 23, 531 (2023)**

**https://doi.org/10.1186/s12884-023-05855-4**

Following publication of the original article [1], the authors reported an error in Table 3 in PDF proof. Table 3 in the PDF version contains incomplete details, but HTML version contains complete details.

The original article has been corrected.
